# Eukaryotic initiation factors: central factor associating mRNA translational plasticity during neuropathic pain progression

**DOI:** 10.3389/fneur.2025.1566205

**Published:** 2025-07-30

**Authors:** Xinshuo Li, Haibo Zhan, Xindan Zhang, Jiayi Li, Xiangrui Li, Xihua Lu, Changhong Miao, Chunli Zhou, Zhen Zhang

**Affiliations:** ^1^Department of Anesthesiology, The Affiliated Cancer Hospital of Zhengzhou University & Henan Cancer Hospital, Zhengzhou, Henan, China; ^2^Department of Anesthesiology, Huadu District People’s Hospital of Guangzhou, Huadu Institute of Medical Sciences, Guangzhou, Guangdong, China; ^3^The 1st Clinical Department, China Medical University, Shenyang, Liaoning, China; ^4^Department of Anesthesia and Perioperative Medicine Zhongshan Hospital, Fudan University, Shanghai, China; ^5^Department of Anesthesiology, Xiangyang Central Hospital, Affiliated Hospital of Hubei University of Arts and Science, Xiangyang, Hubei, China

**Keywords:** neuropathic pain, eIF4E, eIF2α, eIF4G, plasticity, translation

## Abstract

Neuropathic pain causes plasticity in the nervous system, which is often associated with altered protein synthesis. Proteins are the key executors of cellular functions, and their alteration is closely related to the occurrence of neuropathic pain. Protein synthesis is a finely regulated process involving the interaction of multiple biomolecules. Among them, the eukaryotic translation initiation factors (eIFs) are a group of key regulatory proteins that control the initiation phase of protein translation and thus influence the rate and type of protein synthesis. Recent studies have shown that the eIFs are involved in the regulation of neuropathic pain regulating translation through phosphorylation and affecting the transmission and processing of neuropathic pain signals. Among them, eIF4E and eIF2α, as core initiation factors, changes in their expression and activity are closely associated with various neuropathic pain. This review aims to summarize the evidence for the involvement of the eIFs, especially eIF4E and eIF2α, in pain-associated mRNA translational plasticity, and to propose relevant therapeutic approaches. We hope that this review will provide important ideas for future research on the mechanisms of neuropathic pain and new targets for the treatment of neuropathic pain.

## 1 Introduction

Neuropathic pain is a result of injury or dysfunction in the central or peripheral nervous system (1), encompassing a wide range of conditions, from peripheral nerve injuries (e.g., diabetic neuropathy, post herpetic neuralgia) to central nervous system injuries (e.g., spinal cord injury, post-stroke pain). A systematic review of epidemiological studies estimated that the prevalence in the general population is between 7 and 8% (2), highlighting its significance as a major public health concern. Unlike nociceptive pain, which is directly triggered by an external noxious stimulus, neuropathic pain arises from pathological changes in the nervous system itself, such as the abnormal generation and transmission of pain signals. This fundamental distinction in underlying mechanisms makes neuropathic pain more challenging to treat than nociceptive pain. The pathological mechanisms of neuropathic pain are significantly influenced by changes in nervous system plasticity, including both peripheral and central sensitization (3). These plastic changes are largely dependent on gene transcription and protein translation processes (4). While transcription plays a significant role in regulating gene expression, in the context of neuropathic pain, gene expression alterations are primarily regulated at the translational level (5). Consequently, investigating translation processes can provide more critical insights into the plastic changes occurring within the nervous system.

Nerve tissue damage is closely related to the dysregulation of protein translation in the tissue. In diabetic peripheral neuropathic pain, chemotherapy-induced neuropathic pain, post-herpetic neuralgia (PHN) and neuropathic pain due to nerve injury, dysregulated protein translation leads to the remodeling of neuronal function and structure. Translational dysregulation affects synaptic plasticity, cellular energy metabolism, and integrative stress responses through many signaling pathways and plays a critical role in the development and maintenance of neuropathic pain (6). Specifically, in the context of neuropathic pain, protein translation regulates the synthesis of pain-related proteins, including altered expression or distribution of ion channels, increased levels of inflammatory mediators and neurotrophic factors, and elevated kinase activity associated with translational control. In several experimental studies in animal models, it has been shown that activation of ion channels, release of neurotrophic factors and inflammatory mediators leads to alterations in neuronal excitability, which in turn leads to neuronal plasticity (7–10). Notably, the accuracy of gene expression is not only dependent on transcriptional regulation, but also critically constrained by the dynamic balance of translation. In the case of neuropathic pain, this dysregulation further exacerbates the vicious cycle of neuronal hyperexcitability and the inflammatory microenvironment through spatio-temporal-specific proteomic remodeling. Overall, protein translation dysregulation is a key factor controlling the development and maintenance of neuropathic pain, which not only affects neuronal function and signaling but is also closely related to neuroinflammatory responses and neuroplasticity.

Gene expression is tightly regulated at both the transcriptional and translational levels, and although protein levels are mainly determined by the levels of their encoding mRNAs, recent studies have found that transcriptional levels by themselves are not sufficient for predicting protein levels, suggesting that translational regulation is of great importance in gene expression, which makes changes in mRNA levels not exactly coincide with changes in protein levels (11, 12). In eukaryotes, the cellular abundance of proteins is highly controlled at the level of mRNA translation (13). Translational control has become a crucial mechanism for regulating protein levels in cells (14). This process can be divided into four main stages: initiation, elongation, termination, and the ribosome cycle. Translation is primarily regulated during the initiation phase, which represents the rate-limiting step in the translation cascade. The eukaryotic initiation factor (eIF) family are a group of proteins widely found in eukaryotes that affect protein synthesis by regulating translation initiation. Dysregulation of translation can lead to aberrant protein expression, which is considered one of the key factors in the development of various human diseases (15–17). Recently, significant progress has been made in understanding the role of the eIFs in pain regulation, yet no review has systematically summarized and provided an in-depth analysis of the pathophysiological mechanisms through which the eIFs contributes to neuropathic pain. This review aims to summarize the relevant biological features and functions of the eIFs, as well as the progress of research on neuropathic pain, with a particular focus on the role of the eIFs in the regulation of mRNA translation and its involvement in neuropathic pain plasticity.

## 2 Biological traits of the eIFs

At least 15 eIFs are involved in translational regulation, many of which are large multi-subunit complexes (18). This underscores the complexity and sophistication of the eukaryotic translation initiation process. Each translation initiation factor has its own specific properties and roles, including codon recognition, ribosome binding, interaction with other proteins, cellular stress responses, and involvement in a variety of diseases (19–21). Some members of the eIFs have been shown to regulate translational processes in the nervous system, thereby influencing neurotransmission and nociceptive modulation (22). Dysfunction of eIFs are believed to contribute significantly to the development of neuropathic pain, particularly by modulating neuroplasticity and the inflammatory response via translation, which, in turn, affects the chronicity and persistence of pain (23–25).

### 2.1 eIFs and translation initiation

**Figure 1:**
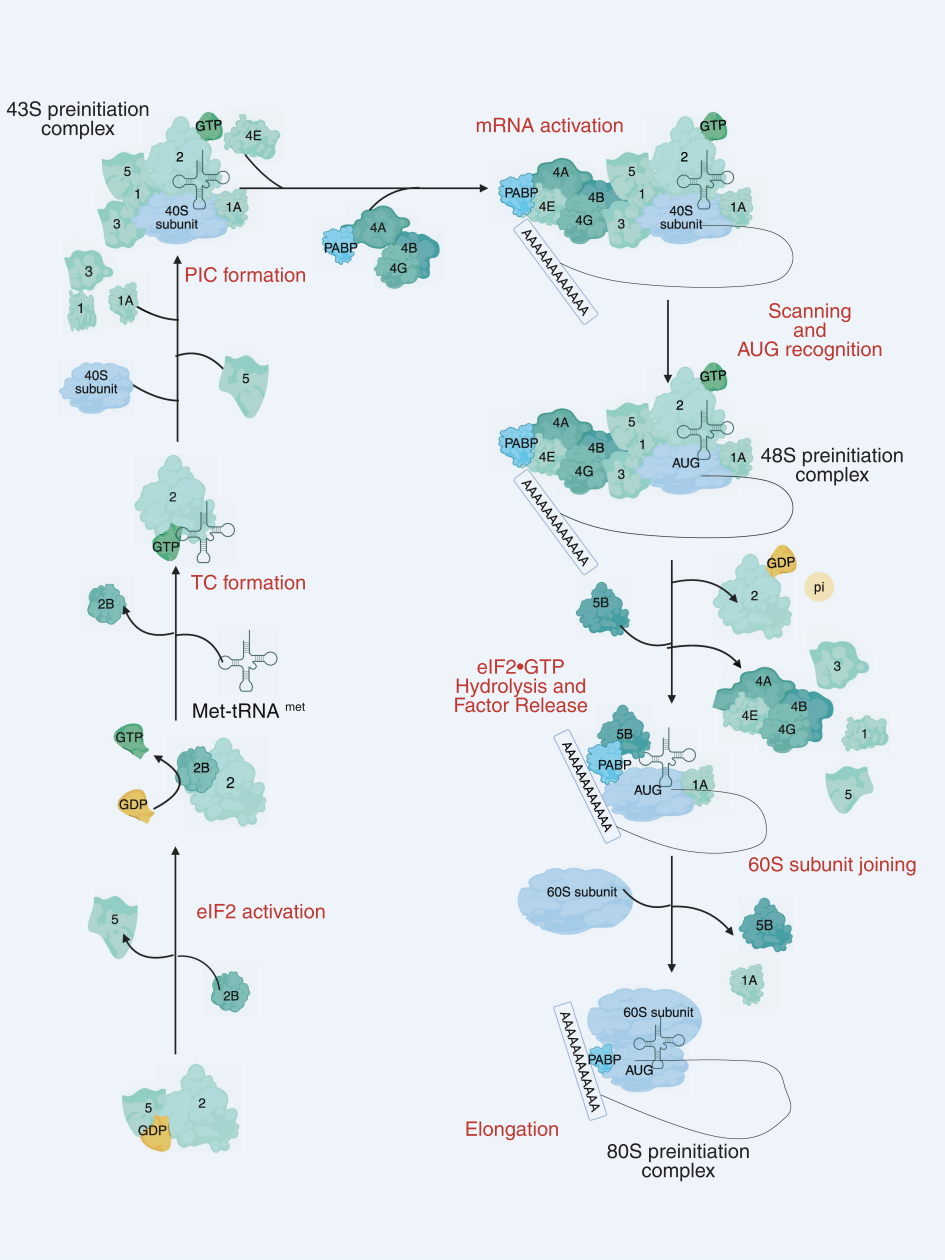
The main steps of translation initiation in eukaryotes. Initiation begins with the assembly of the 43S preinitiation complex (PIC), consisting of the 40S small ribosomal subunit, eIF1, eIF1A, eIF3, eIF5, and the ternary complex eIF2-GTP-Met-tRNAi. The PIC is recruited to the 5 cap of the mRNA by the eIF4F complex (eIF4E, eIF4G, and eIF4A) and eIF4B. Circularization of the mRNA is promoted by interaction between eIF4G and PABP. The mRNA is scanned in a 5′ to 3′ direction until a start (AUG) codon is recognized, triggering the release of eIF1, inorganic phosphate (Pi), eIF5, and eIF2-GDP. The joining of the 60S ribosomal subunit to the PIC and release of several initiation factors, including eIF1A, is catalyzed by eIF5B, leading to the formation of the elongation-competent 80S ribosome. Numbers represent the corresponding eIFs.

The eIFs consist of several key components, including eIF1, eIF1A, eIF2, eIF2B, eIF3, eIF4A, eIF4B, eIF4E, eIF4G, eIF4H, eIF5, eIF5B, and eIF6 (see Table 1 for details). Translation initiation is marked by the binding of eIF1, eIF1A, eIF3 and eIF5 to the small subunit of the 40S ribosome. Subsequently, a ternary complex is formed, consisting of eIF2, guanosine 5′-triphosphate (GTP), and the methionine initiator tRNA (Met-tRNAiMet), which bind to create the 43S pre-initiation complex. This complex serves as the foundation for the initiation process, facilitating the assembly of the translation machinery. At the same time, eIF4F, a complex composed of the cap-binding protein eIF4E, the RNA helicase eIF4A, and the large scaffolding protein eIF4G, binds to the 5′ end of the mRNA and recruits the 43S ribosomal subunit to form the 48S initiation complex (48S). The 48S complex then scans along the 5′ untranslated region (UTR) until it encounters the start codon AUG, where another molecule of eIF4A, along with the cofactor eIF4B or its homolog eIF4H, unwinds the secondary structure of the mRNA downstream of the 48S. During this scanning process, eIF5 stimulates irreversible GTP hydrolysis, leading to the release of the eIF2-GDP complex. The GDP bound to eIF2 must be exchanged for GTP in a guanine nucleotide exchange reaction catalyzed by eIF2B before eIF2 can re-form the ternary complex. The scanning process culminates in the release of most eIFs upon recognition of the start codon by the 48S complex. The subsequent binding of eIF5B facilitates the joining of the large 60S subunit, leading to the formation of the 80S initiation complex, thereby completing the final step of translation initiation (26) (see Figure 1). For a more detailed summary of the functions of these translation initiation factors, see Table 1. The eIFs orchestrate the assembly of the ribosomal machinery, facilitate mRNA recruitment, and ensure start codon to guarantee accurate and efficient protein synthesis. Dysregulation of specific components of the eIFs, either through mutation or via the enhancement of aberrant signaling pathways, can significantly disrupt these critical stages. Such disruptions may result in either hyperactivation or inhibition of translation, leading to aberrant protein expression. These translational abnormalities are closely associated with the onset and progression of many diseases, including neurodegenerative diseases, immunodeficiencies, metabolic disorders, and cancer (27–30). Therefore, understanding the exact role and regulatory mechanisms of the eIFs are crucial for elucidating the molecular basis of various pathologies and developing targeted therapeutic strategies.

**Table 1:** Functions of the eIFs in translation initiation, associated diseases, and relevant references.

eIF*	Function	Ref	Diseases and references	
eIF1	Regulator of start codon recognition. Before the recognition of AUG, eIF5 is prevented from binding to key sites in the PIC required to trigger downstream events.	(19)	Hepatocellular carcinoma (167) Colorectal cancer (168)	
eIF1A	Stimulation of PIC assembly and scanning. Fidelity of start codon selection.	(169)	Uveal cancer (170, 171) Thyroid cancer (170, 171) Ovarian cancer (170, 171) Melanoma (167, 170) Epithelial carcinoma (170)	
eIF2	eIF2 can Bind GTP and Met-tRNA and transfer Met-tRNA to the 40S ribosomal subunit.	(160)	Adenomyosis (172) Alzheimer’s disease (168) Primary sclerosing cholangitis (22) Alzheimer’s disease (173)	
eIF2B	eIF2B is the guanine nucleotide exchange factor for eIF2.	(174)	Melanoma (167) Vanishing white matter (175, 176) Typical endometrial hyperplasia (172) Oropharyngeal cancer (167) Non - Hodgkin lymphoma (170) Alcoholic liver disease (177) Post- operative cognitive dysfunction (178)	
eIF3	eIF3 can bind the 40S subunit and prevent binding of the 60S subunit to the 40S subunit and stimulate 43S PIC assembly.	(179)	Cervical cancer (167) Colorectal cancer (168) Bladder cancer (167) Prostate cancer (167) Osteosarcoma (167) Hepatocellular carcinoma (167) Glioma (167) Ovarian cancer (170) Endometriosis (172) Non - small cell lung cancer (170) Pancreatic cancer (167, 170) Melanoma (167, 170) Herpes simplex virus (180) Rheumatoid arthritis (181) Poliomyelitis (180) Parkinson’s disease (180)	
eIF4A	eIF4A is a DEAD-box RNA helicase.	(182, 183)	Osteosarcoma (184) acute myeloid leukemia (185) breast cancer (153) lung cancer (186) pancreatic cancer (187) Melanoma (188)	
eIF4B	eIF4B stimulates the eIF4A helicase to unwind secondary structures in mRNA, and is a downstream target of mTOR.	(189, 190)	Breast cancer (167) Melanoma (167) Non - small cell lung cancer (170)	
eIF4E	eIF4E binds to the 5′ cap of mRNA, enabling its recognition by the translation initiation complex and facilitating the assembly of the ribosome for protein synthesis.	(191)	prostate cancer (192) Gastric cancer (193) Glioma (194) head and neck cancers (195) breast cancer (196) acute myeloid leukemia (197) Ovarian Cancer (198) Psoriatic (199)	
eIF4G	eIF4G is a scaffolding protein that links the mRNP to the PIC, interacting with RNA, eIF4E, eIF4A, and eIF3 among others.	(191)	Hepatocellular Carcinoma (200) breast cancer (201)	
eIF4H	eIF4H increases helicase activity of eIF4A. eIF4H gene is absent in a neurodevelopmental disorder called Williams syndrome.	(202, 203)	Lung adenocarcinoma (167) Glioblastoma multiforme (204)	
eIF5	eIF5 promotes translation initiation fidelity through interactions with the 40S, eIF1A and eIF2.	(205)	Lung cancer (170) Oxidative stress (206)	
eIF5B	eIF5B is a GTPase that stimulates 60S subunit joining.	(20)	Glioblastoma multiforme (170) Lung cancer (170) Hepatocellular carcinoma (170) Hepatocellular carcinoma (207)	
eIF6	Blocks the assembly of 60S and 40S ribosomal subunits in the cytoplasm. Binds to 60S ribosomes in the nucleolus.	(208)	Leukemia and lymphoma (167) Atherosclerosis (209)	

### 2.2 Upstream signaling pathways involved in translational regulation by the eIFs

Three major signaling pathways cooperatively regulate the initiation of translation: the mTOR complex 1 (mTORC1) pathway, the mitogen-activated protein kinase (MAPK) pathway, and the integrated stress response (ISR) pathway. In the mTORC1 pathway, mTORC1 phosphorylates eIF4E-binding protein 1 (4E-BP1), impairing its binding to eIF4E upon phosphorylation and thereby releasing eIF4E for cap-dependent translation. In the MAPK cascade, activation of MAPK by mitogenic or stress signals induces phosphorylation of mitogen-activated protein kinase-interacting kinases 1/2 (MNK1/2), which subsequently phosphorylate eIF4E at Ser209 to enhance its binding affinity to mRNA (31). The ISR pathway, in response to cellular stress, also phosphorylates eIF2α, leading to a reduction in general translation initiation but preferential translation of stress-related mRNAs, including ATF4. This pathway can further influence eIF4E phosphorylation indirectly through downstream signaling cascades, contributing to the regulation of translation under stress conditions (32).

#### 2.2.1 The mTOR signaling pathway

**Figure 2:**
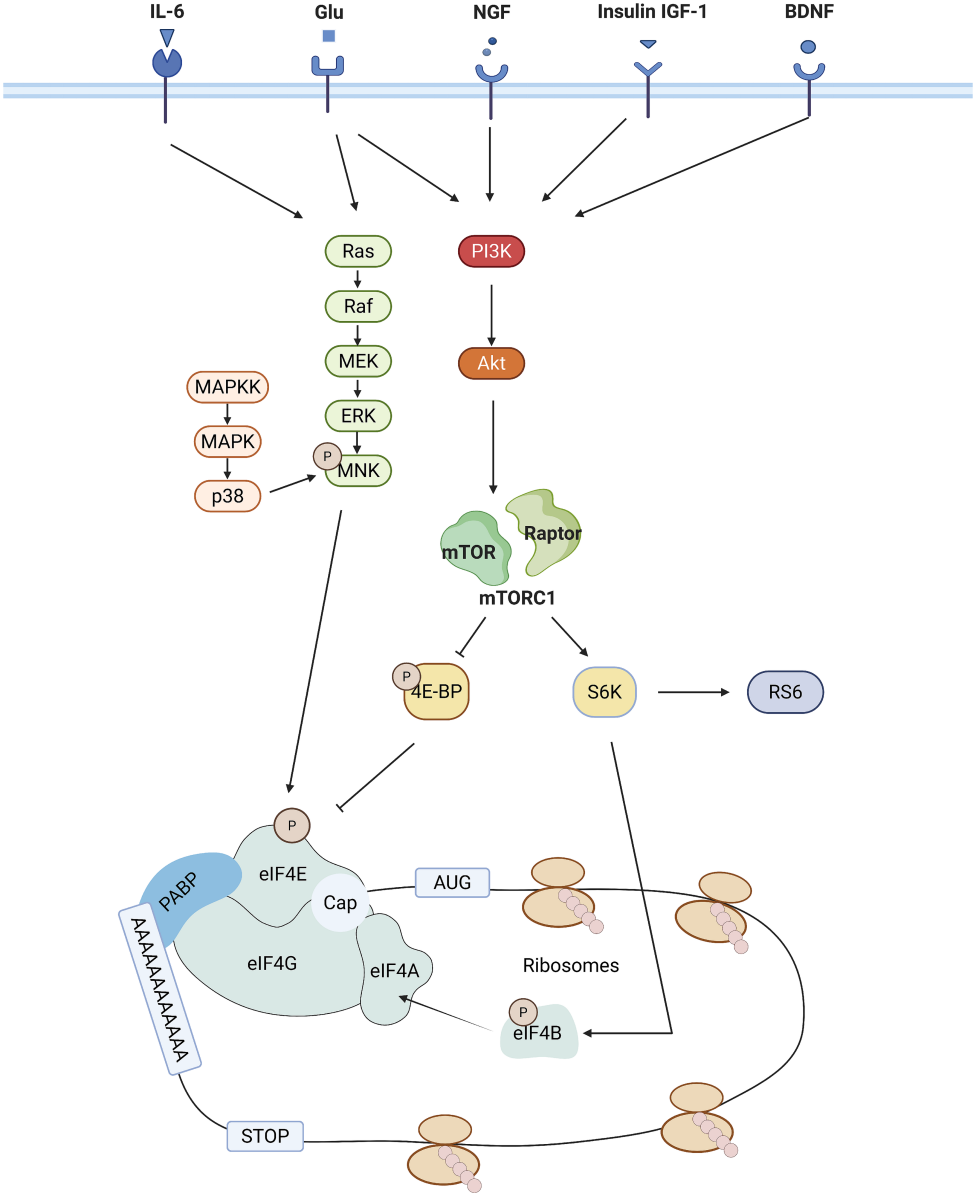
Schematic representation of the major signaling pathways that regulate eIF4E activity. mTORC1 and MNK signaling pathway activity is sequentially regulated by a variety of external [tyrosine receptor kinase a (trkA) and trkB, receptors from the insulin receptor family [IR, insulin-like growth factor-1 receptor (IGF1R), epidermal growth factor receptor (EGFR)], metabotropic glutamate and N-methyl-D-aspartate (NMDA) receptors] and internal cues [cellular energy status (via AMPK)]. In addition, the activation of mTOR is also mediated by the phosphorylation downstream of the PI3K/AKT pathway. Activation of mTOR is followed by phosphorylation of eIF4B by S6 K1, which induces binding of eIF4B to eIF4A, an event that enhances eIF4A deconjugating enzyme activity. eIF4E is a downstream effector of the mTOR and MAPK signaling pathways. A key step in translation initiation is the binding of eIF4E to the mRNA cap. eIF4E mediates the formation of the eIF4F complex on the structure of the mRNA cap. eIF4F complexes include, in Alzheimer’s disease to eIF4E, eIF4G (scaffolding proteins) and eIF4A (deconjugating enzymes).

The mTOR signaling pathway is a key regulatory hub for cell growth, metabolism and survival, coordinating multiple cellular functions including cell growth, proliferation, protein synthesis and energy metabolism by integrating external signals such as hormones (e.g., insulin), growth factors, oxygen levels and nutrients (e.g., amino acids; see Figure 2) (33–36). As a major downstream effector of the mTOR pathway, the activity of eIF4E can be regulated by signals that activate this signaling pathway, and eIF4E is able to respond to these diverse stimuli in a synchronized manner, so eIF4E can be said to be a central regulator of this signaling pathway. The mTOR pathway consists of two distinct complexes, mTORC1 and mTORC2, which differ in their sensitivity to rapamycin and control separate biological processes (37).

Specifically, mTORC1 functions as a key sensor of external stimuli, regulating a variety of downstream targets, including 4E-BP1 and S6 kinase. Upon activation, mTORC1-mediated S6K phosphorylates both eIF4B and ribosomal protein S6, thereby modulating translation rate. In its non-phosphorylated form, 4E-BP1 binds to eIF4E and prevents the formation of the eIF4E-eIF4G complex, thereby inhibiting cap-dependent translation. Upon activation of mTORC1, 4E-BP1 is phosphorylated, releasing eIF4E, which can then bind to eIF4G, initiating the translation of eIF4E-dependent mRNAs (38–40). Dysregulated mTOR signaling has been associated with a variety of human diseases. In cancer, hyperactivation of mTOR drives protein translation and anabolism, supporting cancer cell proliferation (41, 42). In the nervous system, particularly in neuropathic pain, it involves the resynthesis of proteins in injurious conduction pathways (43). In neurodegenerative diseases such as Alzheimer’s disease and Parkinson’s disease, mTOR overactivation inhibits autophagy, leading to the accumulation of pathological proteins (e.g., *β*-amyloid, *α*-synuclein) (44, 45). In addition, dysregulated mTOR signaling is strongly associated with obesity, insulin resistance, type 2 diabetes mellitus, and non-alcoholic fatty liver disease (NAFLD) (46–48), and insulin and estrogen signaling through mTOR and PI3K synergistically regulate autophagy and mitochondrial metabolism, whose disruption contributes to metabolic diseases (49).

#### 2.2.2 The MAPK signaling pathway

MAP kinases belong to the serine–threonine protein kinase family and have been widely conserved throughout evolution to regulate various physiological processes (50). Eukaryotic cells utilize multiple distinct MAPK signaling pathways, with the most well-studied groups in mammals being ERK1/2, JNK, and p38 isoforms. MAP kinases regulate a wide range of physiopathological processes, including cell growth, differentiation, stress responses, and apoptosis, through a phosphorylation cascade involving three upstream kinases: a MAP kinase kinase kinase (MAPKKK), a MAP kinase kinase (MAPKK), and a MAP kinase (MAPK) (51). MNK1/2 are downstream signaling molecules in this cascade, encoded by two genes, mnk1 and mnk2. MNK is activated by phosphorylation of threonine residues in its activation loop, catalyzed by ERK or p38 MAPK (31, 52). The MAPK pathway is involved in a variety of disease processes (53–55), and its regulatory role is often exerted through the modulation of eIFs functions. Upon receiving a foreign stimulus (53), eIF4E activity is further regulated by MNK1, which is primarily responsible for inducing eIF4E phosphorylation (see Figure 2) (31). eIF4E phosphorylates eIF4E at serine 209. This is thought to be the only post-translational modification of eIF4E, and the phosphorylation of eIF4E enhances its ability to stimulate mRNA translation (56). However, it has been demonstrated that MNK1 does not interact directly with eIF4E, instead, it mediates this process by binding to the C-terminal region of eIF4G (57). Therefore, it is likely that eIF4E phosphorylation occurs during or after the assembly of the eIF4F complex. These signaling pathways (p38, ERK, and JNK) have all been reported to play an important role in neuropathic pain and can be activated in neurons or glial cells (58–62). Consequently, the involvement of MAPK signaling in the regulation of pain is most likely mediated through the regulation of eIFs-controlled translation. There is growing evidence that dysregulation of the MAPK pathway is associated with a variety of diseases, and it is a key factor in carcinogenesis, involving cell proliferation, invasion, and metastasis (63). For example, activation of p-ERK drives tumor angiogenesis by enhancing the expression of pro-angiogenic factors such as VEGF (64), and ERK1/2 promotes the translation of oncogenic proteins such as c-Myc by phosphorylating eIF4E, a mechanism that has been demonstrated in several cancers (65). JNK and p38 pathways are particularly prominent in neuroinflammation and neuropathic pain, where they are activated in neurons and glial cells following nerve injury. These kinases drive the expression of pro-inflammatory cytokines (e.g., TNF-*α*, IL-1β) and enhance the sensitivity of injury sensory circuits, leading to chronic pain (66–69). Furthermore, p38 MAPK is a central mediator of inflammatory diseases such as rheumatoid arthritis and inflammatory bowel disease, mediating joint inflammation and bone destruction by promoting the production of IL-1β, TNF-α and IL-6 (70).

#### 2.2.3 The ISR signaling pathway

The Integrated Stress Response (ISR) is an evolutionarily conserved intracellular signaling network that helps cells cope with a variety of adverse stimuli, including intrinsic stresses such as endoplasmic reticulum (ER) stress and extrinsic stresses like glucose and amino acid deficiencies, hypoxia, viral infections, and reactive oxygen species (ROS) (71). At the core of the ISR is the phosphorylation of eIF2α, which is carried out by one of four kinases (GCN2, PERK, HRI, and PKR) in response to various stressors. This phosphorylation results in the binding of eIF2α to eIF2B, thereby preventing the GDP-to-GTP exchange and consequently reducing the activity of the ternary complex. As a result, this globally inhibits translation initiation, and the decreased concentration of the ternary complex also impacts the start codon selection of regulatory mRNAs that have multiple start sites, such as ATF4 (32, 72). PKR, GCN2 and PERK are key kinases regulating eIF2α phosphorylation, each of which responds to different stress signals. In the context of pain, PKR activation is closely associated with neuroinflammation and central sensitization and has been shown to modulate the expression of inflammatory cytokines in glial cells, which in turn amplify pain signals (73–75). GCN2 is able to participate in the modulation of mitochondrial morphology and function, which has been associated with a variety of neurodegenerative disorders (76), and GCN2 activation induces eIF2α phosphorylation, leading to stress granule (SG) formation and cell death, a process in neurodegeneration that may affect pain maintenance (77). PERK, on the other hand, is a key kinase that responds to endoplasmic reticulum stress and is currently the most studied kinase in neuropathic pain. Endoplasmic reticulum stress is usually caused by the accumulation of misfolded proteins in the endoplasmic reticulum. Activation of PERK phosphorylates eIF2α and inhibits global protein synthesis to protect cells from endoplasmic reticulum stress. More importantly, PERK is involved in the regulation of apoptosis and inflammatory responses by regulating the transcription of genes such as ATF4 and CHOP (78). In macrophages and glial cells, the PERK-eIF2α pathway has been shown to promote pro-inflammatory polarization and participate in neuroinflammatory processes, which have important implications for the development of neuropathic pain (79–81).

## 3 The eIFs regulates neuropathic pain

The mechanisms underlying neuropathic pain are highly intricate, encompassing a multitude of dynamic molecular and cellular processes. These include altered ion channel function, the initiation and maintenance of inflammation response, and synaptic plasticity. In recent years, it has been demonstrated that multiple members of the eIFs play a pivotal role in regulating these essential mechanisms. The following discussion will provide a detailed account of the involvement of the eIFs in the regulation of neuropathic pain through the aforementioned molecular signaling pathways at multiple levels of biological processes, thereby offering new insights into the understanding and treatment of neuropathic pain.

### 3.1 Involvement of eIF in ion channel regulation

The expression and distribution of ion channels in neurons is critical for the formation and maintenance of membrane excitability. Members of the eIFs play a role in the regulation of neuropathic pain by modulating the expression and function of ion channels, includingcalcium (Ca^2+^), potassium (K^+^) channels and Transient receptor potential vanilloid (TRPV). Many studies have shown that there are other channels whose expression mechanisms are also altered in neuropathic pain; however, these mechanisms are not yet known, and there is still a great deal of tremendous research potential in this area.

#### 3.1.1 eIF4E and the regulation of calcium channels

eIF4E regulates the expression of calcium channel isoforms such as Cav2.2 (N-type calcium channel) in particular through selective translation (82). sig-1Rs (ER) chaperones associated with neuropathic pain and enriched in the dorsal root ganglia (DRG) regulate Cav2.2 (N-type VGCC) through an upstream mechanism by decreasing the translation of Cav2.2 mRNA, which reduces voltage-gated protein levels of Cav2.2, which is involved in the onset of DRG neuronal excitability. The promoter of 4E-BP1 can bind Sig-1R, leading to the upregulation of 4E-BP1. The mutual binding of the two prevents eIF4E from initiating the mRNA translation of Cav2.2. Dysfunction of voltage-gated calcium channels leads to neuropathic pain (82). Similarly, Jeevakumar et al. demonstrated that increased excitability of injury receptors in neuropathic pain is due to MNK-eIF4E-mediated translation of factor-upregulated voltage-gated calcium channels (83). In addition, internalization of the L-type calcium channel (LTC) CaV1.2 is mediated by binding to eIF3E (a subunit of eIF3), which is widely present in neurons, and in this way we can speculate that eIF3 molecules may also be involved in the regulation of neuropathic pain (84).

#### 3.1.2 Role of eIF4G2 and potassium channels

Kim et al. found that a decrease in Kv current in the DRG in the chronic compression injury (CCI) model was associated with elevated nerve excitability (85). Zhang et al. (80) demonstrated that, after peripheral nerve injury, there is an increase in both mRNA and protein expression of eIF4G2. Kv1.2 mRNA contains a 5′ cap structure, and eIF4G2 acts as a general inhibitor of cap-dependent mRNA translation. Consequently, eIF4G2 may downregulate the expression of Kv1.2 by inhibiting its mRNA translation, leading to increased neural excitability in the DRG and significantly enhanced neural responses to mechanical, thermal, and cold stimuli, resulting in injurious hypersensitivity. This also suggests that the reduction in Kv1.2 expression is a specific response to eIF4G2.

However, recent studies have revealed a role for eIF4G2 in an alternative cap-dependent translation initiation pathway that is distinct from its traditional function as a general repressor. It has been shown that eIF3d and eIF4G2 are able to mediate a cap-dependent translation initiation mechanism that is independent of eIF4E (86, 87). In this mechanism, a specific subset of mRNAs can undergo cap-dependent translation despite the isolation of eIF4E, by preferentially utilizing eIF3d and eIF4G2, and eIF4G2 serves as a strong binding partner of eIF3d (88). Furthermore, Roiuk et al. demonstrated through immunoprecipitation studies that during mTORC1 inhibition, eIF3d-dependent mRNAs decreased their binding to eIF4E, but their binding to eIF3d and eIF4G2 increased. These mRNAs are able to ‘release’ eIF4E and bind to eIF3d and eIF4G2, thereby facilitating their translation (88). Through their interaction, eIF3d and eIF4G2 are able to mediate a previously unexplored form of cap-dependent translation initiation, which has been demonstrated during epithelial-mesenchymal transition (87). Further research has also revealed that in regulatory T cells, eIF3d and eIF4G2-mediated translational switches are critical for maintaining cell survival and invasiveness (87, 89). Notably, eIF3d and eIF4G2 are able to act synergistically to initiate a cap-dependent translational pathway that is independent of eIF4E, a mechanism that may play a critical role in neurological responses, especially following nerve injury. For example, in the setting of neuroinflammation and injury, specific mRNAs may be preferentially translated through this pathway, thereby enhancing neuronal excitability and leading to the persistence of pain.

#### 3.1.3 Role of eIF2α and eIF4E with TRPV

Transient receptor potential vanilloid type 1 (TRPV1): TRPV1 belongs to the transient receptor potential (TRP) family, which is highly sensitive to thermal and chemical stimuli (90). It is expressed in somatic and visceral injury-sensing neurons, neurons of the central nervous system, and cells of the immune system (91). Furthermore, TRPV1 is involved in various types of inflammatory pain, neuropathic pain, and other pain modalities (92). The activation of TRPV1 can regulate Ca2 + influx, which in turn triggers the release of neuropeptides and excitatory amino acids from nerve endings, ultimately causing the formation of nociception in the cerebral cortex (93). Amaya et al. (94) found that TRPV1 is expressed in DRG neurons under inflammatory conditions and is translationally up-regulated through p38 signaling in the DRG. eIF4E is a downstream effector, and phosphorylation of eIF4E has been implicated in the translational regulation of TPRV1 and provides a link between enhanced mRNA translation and neuronal excitability (22). p-eIF2α controls thermal but not mechanical sensitivity by regulating transient TRPV 1 activity. In models of chronic inflammation, eIF2α phosphorylation is induced in primary nociceptor and it contributes to inflammatory pain hypersensitivity (95).

### 3.2 Inflammation response

The inflammatory response plays a crucial role in the development and maintenance of neuropathic pain, involving complex interactions among cytokines, glial cells, immune cell infiltration, and oxidative stress. Key cellular contributors include immune cells—such as macrophages, neutrophils, dendritic cells, natural killer (NK) cells, and T cells—as well as glial cells including microglia, astrocytes, and satellite glial cells (96, 97). Following nerve injury, monocyte-derived macrophages infiltrate the DRG, where they integrate with resident macrophages and release pro-inflammatory, pronociceptive mediators, leading to neuronal sensitization (98–101). Similarly, T cells infiltrate injured nerves, the DRG, and spinal cord, promoting the transition from acute to chronic pain by releasing cytokines and chemokines (102). Glial cell activation also plays a central role: microglia are activated through CX3CL1–CX3CR1 signaling; astrocytes undergo endoplasmic reticulum (ER) stress; and satellite glial cells, activated by tumor necrosis factor *α* (TNF-α), enhance nociceptive neuron excitability and contribute to mirror pain (21, 103, 104). These cells release inflammatory mediators such as TNF-α, interleukin 1 (IL-1), interleukin 6 (IL-6), and nerve growth factor (NGF), which directly stimulate sensory neurons and DRG neurons, increasing their excitability and promoting pain transmission (105–107). The eukaryotic translation initiation factor (eIF) family regulates these inflammatory responses at the translational level. For example, IL-6 and NGF converge on the eIF4F complex via the ERK/mTOR signaling pathway, promoting proteolysis of primary sensory neurons and axons, contributing to sustained mechanical hypersensitivity, a process that can be attenuated by inhibitors of general and cap-dependent translation (107–109). In macrophages, paclitaxel induces the release of HMGB1 through the ROS/p38MAPK/NF-κB pathway, where eIF4E, a downstream effector, likely modulates the resulting neuronal excitability via RAGE and CXCL12/CXCR4 signaling (110). Furthermore, ER stress in macrophages activates the PERK-eIF2α pathway, inducing pro-inflammatory polarization and cytokine secretion, thus suggesting that eIF2α also plays a role in macrophage-mediated neuropathic pain (111, 112). In T cells, increased phosphorylation of eIF4E enhances protein synthesis and proliferation, while activation of the PERK–eIF2α–CHOP pathway induces apoptosis and pain signaling (113–115). In glial cells, eIF4E is involved in microglial responses via MAPK signaling (116), and in astrocytes, the PERK-eIF2α pathway regulates protein synthesis under ER stress, mitigating cellular damage (21). Lastly, in satellite glial cells, type I interferon may suppress pain signaling by inhibiting neurotransmitter release (117). Collectively, these findings underscore the pivotal role of the eIFs in modulating the inflammatory cellular environment and mediators that underlie neuropathic pain, thereby highlighting eIFs as potential therapeutic targets.

### 3.3 Synaptic plasticity

Synaptic plasticity, a fundamental phenomenon in the nervous system, refers to reversible changes in synaptic strength and structure. It is one of the central mechanisms underlying learning, memory, neuroadaptation, and pathological conditions such as pain. Long-term potentiation (LTP), a key form of synaptic plasticity, involves significant alterations in synaptic transmission in the context of spinal cord injury (118). This is evidenced by an enhanced NMDA receptor-mediated excitatory synaptic transmission, alongside a corresponding attenuation of gamma-aminobutyric acid (GABA) receptor- and glycine receptor-mediated inhibitory synaptic transmission. This imbalance promotes central sensitization, where pain signals are amplified at the spinal cord level. Additionally, C-fiber-mediated LTP plays a crucial role in this process, driving substantial alterations in neuronal synaptic plasticity (119, 120), These changes establish a neurobiological foundation for the persistence of neuropathic pain. Dynamic changes in synaptic plasticity reflect alterations in the regulation of protein expression (121) which is influenced by multiple signaling pathways and molecular regulators. In recent years, the role of synaptic plasticity in neurological diseases has garnered increasing attention. Brain-derived neurotrophic factor (BDNF) is a key regulator involved in synaptic plasticity. It may contribute to the production and maintenance of LTP through various mechanisms, thus playing a regulatory role in the onset and progression of neuropathic pain (122). In addition, studies in the DRG have shown that the activation of elongation factor 2 kinase (eEF2K) can induce an integrated stress response (ISR) that regulates the translation and synthesis of BDNF through the GCN2-eIF2α signaling pathway, contributing to neuropathic pain (123). This finding contrasts with the conventional understanding, where activation of the integrated stress response typically leads to inhibition of protein synthesis. However, recent studies by the Campbell lab have demonstrated that phosphorylation of eIF2α by GCN2 plays a key role in regulating BDNF expression. BDNF mRNA contains inhibitory upstream open reading frames (uORFs), which normally prevent BDNF expression. Upon activation of eEF2K-GCN2 and subsequent phosphorylation of eIF2α, ribosomes bypass these uORFs, facilitating BDNF synthesis (123). It is important to note that both eIF4E phosphorylation and eEF2K-GCN2 regulation of BDNF expression are not mutually exclusive mechanisms. Both pathways could contribute to BDNF expression, and this complex regulation may occur in parallel within DRG neurons. Moreover, BDNF synthesis in eukaryotic cells may be regulated by multiple eIFs members involved in this intricate signaling network. One of the fundamental mechanisms of synaptic plasticity is the enhancement of synaptic strength, which relies on the synthesis of new proteins. The resveratrol-induced increase in *α*-amino-3-hydroxy-5-methyl-4-isoxazolepropionic acid receptor (AMPAR) expression results from elevated protein synthesis via regulation of eIF4E (124). This process, regulated by eIF4E, may also be associated with neurodegenerative diseases such as Alzheimer’s disease, where impairment of synaptic plasticity is considered one of the key pathological features of the disease (121). Dysfunction in the eIFs may contribute to the onset and progression of Alzheimer’s disease by regulating synaptic plasticity in neurons through the PI3K-AKT signaling pathway. Thus, the eIFs are not only implicated in the generation and maintenance of pain through mechanisms related to synaptic plasticity but may also play a significant role in the pathology of other neurological disorders. TNF-*α* and IFN are upstream stimulators of eIF4E, and thus, eIF4E can modulate glial cell activity, contributing to neuropathic pain.

## 4 Available eIFs treatments to reduce neuropathic pain

Targeting the eIFs has shown promise in treating a wide range of diseases, particularly in pathological processes associated with aberrant protein synthesis, demonstrating significant efficacy. In the field of pain management, especially in the treatment of neuropathic pain, the strategy of targeting eIFs are gradually emerging as a unique approach with substantial potential. eIFs regulates immunotranslation as well as the translation of neuronal mRNAs, all of which have been implicated in neuropathic pain. Therefore, targeting specific members of the eIFs and related signaling pathways holds promise for inhibiting the aberrant production of pro-inflammatory mediators, modulating neuronal excitability, and alleviating both peripheral and central sensitization of pain. Despite promising preclinical findings, current clinical treatments are not sufficient to fully address neuropathic pain, and extensive research is still underway to identify more effective pharmacological interventions to reduce its impact. We have summarized the current tables relating to treatments targeting eIFs (Table 2).

**Table 2:** Potential treatments for modulating eIFs.

Treatment category	Treatment name	Mechanism of action	Ref
MNK inhibitors	BAY1143269, ETC-206, eFT 508	Inhibit MNK1/2 kinases to reduce phosphorylation of eIF4E (Ser209), modulating specific mRNA translation.	(125, 126, 128, 129)
mTOR inhibitors	Temsirolimus, Everolimus, Torin1, XL388.	Inhibit mTORC1, preventing phosphorylation of 4E-BP1, reducing eIF4E interaction with eIF4G, and inhibiting eIF4E-dependent mRNA translation.	(130, 132, 133)
ISR inhibitors	GCN2 Inhibitors (GCN2iB), PERK Inhibitors (GSK2606414), ISRIB.	Inhibit key ISR components (eIF2α kinases, eIF2B, and eIF2α phosphatases) to modulate stress-induced translation regulation.	(139–141)
Inhibitors of eIF4F complex	4EGI-1, 4E1RCat, 4E2RCat, P20, EGPI-1	Disrupt interaction between eIF4G and eIF4E, inhibiting eIF4F complex formation and cap-dependent translation.	(147–149)
eIF4A inhibitors	PatA, Hipp, RocA.	Inhibit eIF4A activity by blocking RNA binding, affecting translation initiation of specific mRNAs.	(154–156)
Suppression by controlled expression	siRNA, ASOs.	Use siRNAs and ASOs to silence eIF4G2 and eIF4E expression, reducing translation of pain-related genes (e.g., P2X3, TRPV1).	(159–162, 164–166)

### 4.1 MNK inhibitors

MNK inhibitors prevent the phosphorylation of eIF4E at the Ser209 site by inhibiting the activity of MNK kinases 1 and 2, thereby reducing the translation of eIF4E to specific mRNAs. Several drugs are currently under investigation as potential MNK inhibitors, such as cercosporamide, VNLG-152, CGP57380, and so on. However, only three MNK inhibitors have reached the clinical stage: BAY 1143269, ETC-206, and eFT 508, which are primarily used in the treatment of cancer and leukemia (125). BAY 1143269 significantly downregulates the expression of cell cycle proteins mediated by the MNK-eIF4E axis and reduces the release of pro-inflammatory cytokines such as interleukin-1*β* (IL-1β), IL-6, and TNF-*α* (126). ETC-206 has been shown to reduce the MNK-eIF4E-mediated decrease in β-catenin expression, which is particularly relevant for leukemia treatment. Given these effects, targeting the MNK-eIF4E axis could provide a novel therapeutic strategy for neuropathic pain.

In contrast, eFT 508 is a novel MNK1/2 inhibitor with superior selectivity, efficacy, and oral bioavailability. We hypothesize that MNK-eIF4E signaling may regulate the sequential translation of mRNAs encoding proteins that modulate Nav1.8 and membrane trafficking of K + channels, which are involved in post hyperpolarization, or potentially hyperpolarization-activated cyclic nucleotide-modulated (HCN) channels (127). In line with this hypothesis, eFT 508 has demonstrated efficacy in the treatment of neuropathic pain by reversing paclitaxel-induced mechanical hypersensitivity and spontaneous activity of DRG-injured receptors, as well as in preventing neuropathic pain induced by peripheral nerve injury (128, 129).

### 4.2 mTOR pathway inhibitors

The mTOR inhibitor exerts its effect by inhibiting mTORC1 and preventing its phosphorylation of 4E-BP1, which results in the sustained binding of 4E-BP1 to eIF4E. This binding then inhibits the interaction between eIF4E and eIF4G, which in turn reduces the formation of translation initiation complexes and inhibits the translation of eIF4E-dependent mRNAs. Currently, mTOR inhibitors are categorized into four major groups: antibiotic variant mTOR inhibitors (first generation), ATP-competitive mTOR inhibitors (second generation), dual mTOR/PI3K inhibitors (second generation), and other novel mTOR inhibitors (third generation). The first-generation antibiotic rapamycin and rapamycin analogs such as Temsirolimus (CCI-779) and Everolimus (RAD001) inhibit the kinase activity of the mTORC1 complex mainly by binding to FKBP12. Rapamycin has been shown to have a therapeutic effect on neuropathic pain after spinal cord injury (130). However, its use is often associated with incomplete inhibition of the feedback loop, leading to side effects and limiting its efficacy (131). To overcome these limitations, a second generation of mTOR inhibitors has emerged. ATP-competitive mTOR inhibitors, represented by the drugs Torin1 and XL388, have been shown to alleviate neuropathic pain by targeting the ATP site of the kinase domain of the mTOR complex to block mTORC1, thereby reducing the expression of eIF4E levels to affect mRNA translation, and decreasing synaptic plasticity (132, 133). Second-generation mTOR inhibitors do not cause mutations in the FKBP-rapamycin-binding pr (FRB) region; therefore, long-term administration of them will result in fewer side effects. The second-generation inhibitors can act not only in the spinal cord but also in the brain parenchyma such as the insula cortex (132). Dual mTOR/PI3K inhibitors (second-generation) and other novel mTOR inhibitors (third-generation) currently demonstrate significant efficacy only in cancer (134–138). There are no relevant studies available on their role in neuropathic pain, and this topic will not be discussed further.

### 4.3 ISR inhibitors

ISR inhibitors target signaling pathways involved in ISR. These inhibitors can be broadly classified into three categories based on their action on key components of the ISR. Although there are currently few drugs that specifically target the ISR, these drugs can be broadly classified into three categories: targeting eIF2α kinases, targeting eIF2B, and targeting eIF2α phosphatases. These inhibitors are designed to modulate stress-induced translational regulation and attenuate the pathological consequences of prolonged ISR activation. For example, GCN2 inhibitors block GCN2 activation, and GCN2iB has been shown to reduce levels of GCN2 and eIF2α phosphorylation, leading to increased protein synthesis in cells (139). The PERK inhibitor GSK2606414 has demonstrated significant neuroprotective effects (140).

Another class of ISR inhibitors targets eIF2B. These inhibitors act by disrupting the interaction between eIF2α and eIF2B, thereby attenuating the overall inhibition of translation initiation while preserving the selective translation of stress-adaptive mRNAs. For example, Integrated Stress Response Inhibitor (ISRIB) has been shown to specifically block the inhibitory effects of phosphorylated eIF2α. Recent studies have demonstrated that ISRIB attenuates diabetes-induced neuropathic pain in mice and rats (141).

In addition, enhancing phosphatase activity downstream of ISR to reduce P-eIF2α levels is also considered an important approach for ISR inhibition. Quercetin has been shown to promote dephosphorylation of phosphorylated eIF2α and reduce ATF4 expression, thereby improving memory function (142).

In the nervous system, excessive or sustained activation of the ISR may exacerbate neuroinflammation, neuronal death, and synaptic dysfunction, which are major mechanisms of neuropathic pain. Therefore, the study of ISR inhibitors is emerging as a potential therapeutic strategy for the relief of neuropathic pain.

### 4.4 Inhibitors of eIF4F complex

Five inhibitors that disrupt the interaction of eIF4G with eIF4E have been described: 4EGI-1, 4E1RCat (143), 4E2RCat (144), P20 (145) and EGPI-1 (146).

4EGI-1 inhibits the formation of the eIF4F complex by binding to eIF4E and preventing eIF4G recruitment (147). This is the main reason why 4 EGI-1 can disrupt the assembly of the eIF 4F complex and inhibit cap-dependent translation. Experimental evidence indicates that 4EGI-1 induces apoptosis in multiple myeloma cells and inhibits mitochondrial ATP synthesis (148, 149). It can provide a pharmacophore basis to guide the development of inhibitors with higher binding affinity for eIF4E (150). 4EGI-1 has been used to study the eIF4F complex in memory (151) and autism (152). 4E1RCat, 4E2RCat, P20, and EGPI-1. These have still not been used in the nervous system. Zotatifin, a first-in-class eIF4A inhibitor, targets the RNA deconjugating enzyme eIF4A mechanistic studies have shown that it reprograms tumor translation, inhibits translation of Sox4 and Fgfr1, and induces a consistent interferon response in models. Surprisingly, zotatifin significantly synergised with carboplatin, triggering DNA damage and even potentiating the interferon response, thereby suppressing T-cell dependent tumors. This potential efficacy provides a theoretical basis for TNBC therapy (153).

### 4.5 eIF4A inhibitors

eIF4A inhibitors inhibit translation of specific mRNAs by interfering with the translation initiation process through the inhibition of the RNA deconjugase activity of eIF4A. As a key deconjugating enzyme, eIF4A plays an important role in cancer, neurodegenerative diseases, and other pathological conditions caused by aberrant translational regulation. Thus, inhibitors targeting eIF4A have gained considerable attention as potential therapeutic agents.

Currently, Pateamine A (PatA), hippuristanol (Hipp), and Rocaglamide (RocA) are well-known inhibitors of eIF4A. PatA binds to eIF4A and enhances its intrinsic enzyme activity, but it simultaneously inhibits the binding of eIF4A to eIF4G and promotes the formation of a stabilizing ternary complex between eIF4A and eIF4B. These changes in the affinity of eIF4A for its chaperone proteins upon binding to PatA result in the stalling of the initiation complex on mRNA *in vitro* and the induction of stress granule formation *in vivo* (154). Notably, eIF4A itself has been identified as an inhibitor of its own function.

Hipp binds to the carboxy-terminal structural domain of eIF4A, locking it in a closed conformation and inhibiting its RNA binding activity. The degree of mRNA dependence on eIF4A during initiation is influenced by the secondary structure of the mRNA’s 5′ leading region (155). Recently, RocA has been shown to inhibit translation initiation by blocking the phosphorylation of the mRNA cap-binding eukaryotic translation initiation factor eIF4E, while also stabilizing RNA binding within the eIF4F complex. Furthermore, RocA has been reported to protect progenitor cells from chemotherapy-induced cell death, attenuate inflammation in neuronal tissue, and mitigate drug-induced neuronal damage (156). However, the role of eIF4A inhibitors in pain management remains to be thoroughly explored.

### 4.6 Suppression by controlled expression

siRNAs (small interfering RNAs) and antisense oligonucleotides (ASOs) represent two distinct gene silencing technologies that inhibit gene expression through different mechanisms. Both siRNAs and ASOs have the capacity to reduce the expression level of these proteins by targeting the corresponding mRNAs (157, 158). siRNA has also been shown to attenuate CCI-induced mechanical pain and thermally induced hypersensitivity (159). Many animal studies have demonstrated the biological functions of specific genes by reducing their expression levels. For example, the injection of eIF4G2 siRNA into injured DRG inhibited the SNL-induced increase in eIF4G2 expression, mitigated the SNL-induced down-regulation of Kv1.2 in DRG, and alleviated both the development and maintenance of pain (159). For example, siRNA can knock down the expression of several pain-related genes, such as ionotropic nucleotide receptor P2X3 and TRPV1 (160), resulting in analgesia. Since eIF4E mediates the expression of TRPV1 receptor, it is reasonable to hypothesize that siRNAs could inhibit eIF4E-mediated translation of TRPV1 gene thereby reducing neuropathic pain. In addition, siRNAs are also capable of silencing the oncogenic factor eIF4E, leading to a reduction in eIF4E expression, which contributes to anti-cancer effects (161) and other systemic diseases (162). To further optimize the function of siRNAs, encapsulation in nanocarriers such as liposomes, which protect these molecules *in vivo*, has been developed. Several vectors, such as plasmids, have also been used to transport nucleic acids into cells (163). Recently, patisiran (ALN-TTR02) became the first commercially available RNAi-based drug for the treatment of hereditary amyloidogenic transthyretin (hATTR) amyloidosis with polyneuropathy in adults (164).

ASOs are designed to target specific gene sequences. When injected into the DRG, ASOs not only reduce the expression of these genes but also effectively alleviate neuropathic pain (165). ASO therapy targeting eIF4E mRNA has shown promising results in cancer treatment, demonstrating the potential for similar therapeutic benefits in pain management (165). In the DRG, eIF4E plays a critical role in the translation of mRNA, and specific ASOs targeting eIF4E have the potential to reduce eIF4E-mediated mRNA translation in DRG neurons, which may contribute to the alleviation of neuropathic pain. For example, BDNF-targeted ASO given to mice by intrathecal injection can even effectively reduce pain and thus achieve analgesia (123). ASOs are characterized by rapid absorption, prolonged duration of action, and minimal side effects, making them a promising candidate for pain management therapies (158). It is currently a clinical drug for the treatment of neurological disorders. Nusinersen (Spinraza) is the first FDA-approved ASO for the treatment of spinal muscular atrophy (SMA). It significantly improves the survival and motor function of SMA patients by modifying the splicing pattern of the SMN2 gene, which increases the production of functional SMN proteins (166).

## 5 Conclusion and prospect

The eIFs plays a pivotal role in the modulation of neuropathic pain. Comprising key members such as eIF4E and eIF2α, the eIFs exerts a profound impact on multiple levels of the nervous system through its regulation of mRNA translation. Evidence demonstrates that the eIFs controls protein synthesis and influences neuronal function through a variety of signaling pathways, including MAPK-eIF4E and GCN2-eIF2α. These molecules not only regulate the intricate processes of intracellular protein synthesis but are also closely associated with pivotal physiological mechanisms involved in neuropathic pain, such as alterations in ion channels, activation of neuroinflammatory responses, and modulation of synaptic plasticity. In particular, the eIFs modulates neuronal excitability by influencing the expression and function of sodium and calcium ion channels, which subsequently impacts pain transmission. Additionally, the eIFs are implicated in modulating the neuroinflammatory response by activating microglia and macrophages, which release pro-inflammatory factors that further amplify the inflammatory response in the nervous system, thereby exacerbating pain. Furthermore, the eIFs influences synaptic plasticity and modulates synaptic transmission efficiency, which in turn affects the amplification or attenuation of nociceptive signals. Given these mechanisms, therapeutic strategies targeting eIFs molecules show considerable promise for intervening in neuropathic pain. At present, a variety of molecular tools and pharmaceutical agents, including MNK inhibitors, mTOR inhibitors, siRNAs, and ASOs, are being investigated with the goal of reducing pain perception and enhancing patient quality of life by interfering with the function of the eIFs.

Future studies should focus on the following areas: First, clarifying the specific role of eIF4E-dependent translation and its regulatory mechanisms in different types of pain (e.g., inflammatory pain, neuropathic pain, etc.); second, investigating how to precisely target eIF4E without triggering other adverse effects; and third, evaluating the safety and efficacy of existing and potential new drugs for chronic pain patients, while also understanding the interactions between eIF4E and other signaling pathways and how they collectively modulate pain transmission. Finally, it is crucial to explore how eIF4E regulates the sensitization of pain circuits through specific mRNA translations, and how these mechanisms contribute to the development of chronic pain. Through these studies, we expect to develop more effective therapeutic strategies to address the challenges of neuropathic pain.
